# Expanded clinical and experimental use of SOX11 - using a monoclonal antibody

**DOI:** 10.1186/1471-2407-12-269

**Published:** 2012-06-27

**Authors:** Lena Nordström, Ulrika Andréasson, Mats Jerkeman, Michael Dictor, Carl Borrebaeck, Sara Ek

**Affiliations:** 1 Department of Immunotechnology, Lund University, BMCD13, Lund, SE-221 84, Sweden; 2 CREATE Health, Lund University, BMCD13, Lund, SE-221 84, Sweden; 3 Department of Oncology, Lund University, BMCD13, Lund, SE-221 84, Sweden; 4 Department of Pathology, Lund University, BMCD13, Lund, SE-221 84, Sweden

**Keywords:** SOX11, Flow cytometry, Immunohistochemistry, Transcription factor, B cell lymphoma diagnostics

## Abstract

**Background:**

The transcription factor SOX11 is of diagnostic and prognostic importance in mantle cell lymphoma (MCL) and epithelial ovarian cancer (EOC), respectively. Thus, there is an unmet clinical and experimental need for SOX11-targeting assays with low background, high specificity and robust performance in multiple applications, including immunohistochemistry (IHC-P) and flow cytometry, which until now has been lacking.

**Methods:**

We have developed SOX11-C1, a monoclonal mouse antibody targeting SOX11, and successfully evaluated its performance in western blots (WB), IHC-P, fluorescence microscopy and flow cytometry.

**Results:**

We confirm the importance of SOX11 as a diagnostic antigen in MCL as 100% of tissue micro array (TMA) cases show bright nuclear staining, using the SOX11-C1 antibody in IHC-P. We also show that previous reports of weak SOX11 immunostaining in a fraction of hairy cell leukemias (HCL) are not confirmed using SOX11-C1, which is consistent with the lack of transcription. Thus, high sensitivity and improved specificity are demonstrated using the monoclonal SOX11-C1 antibody. Furthermore, we show for the first time that flow cytometry can be used to separate SOX11 positive and negative cell lines and primary tumors. Of note, SOX11-C1 shows no nonspecific binding to primary B or T cells in blood and thus, can be used for analysis of B and T cell lymphomas from complex clinical samples. Dilution experiments showed that low frequencies of malignant cells (~1%) are detectable above background using SOX11 as a discriminant antigen in flow cytometry.

**Conclusions:**

The novel monoclonal SOX11-specific antibody offers high sensitivity and improved specificity in IHC-P based detection of MCL and its expanded use in flow cytometry analysis of blood and tissue samples may allow a convenient approach to early diagnosis and follow-up of MCL patients.

## Background

The transcription factor SOX11 has recently been identified as a diagnostic, prognostic and/or functional antigen in a variety of cancers including mantle cell lymphoma (MCL) [1-6], epithelial ovarian cancer (EOC) [7,8] and gliomas [9]. This transcription factor is a member of the SOX protein family, which is characterized by a conserved high mobility DNA binding domain (HMG) [9]. Of major clinical interest, the expression of SOX11 in non-malignant tissues is limited to immature neurons [10] and embryonic [11-13] and neural development [14,15].

This tissue-restricted expression facilitates specific identification of malignant cells in clinical samples and has enabled the use of this target for specific diagnosis of MCL, as shown in a number of studies [1-6]. SOX11 expression has also been reported in a fraction of other T and B-cell lymphomas, including B- and T-cell lymphoblastic lymphomas and a subset of Burkitts lymphoma [3,16], but the most common B-cell lymphomas lack SOX11 expression. Thus, using SOX11 as a diagnostic marker for MCL avoids misclassification of morphologically similar CD5^+^ marginal zone lymphomas (MZL) or CD23^-^ chronic lymphocytic leukemia (CLL). In addition, several studies suggest that SOX11 is a prognostic indicator in MCL, although it remains to be confirmed whether negative cases identify a rare indolent subtype [4,17] or, conversely, patients with a shorter survival [2]. Furthermore, in EOC, SOX11 has prognostic significance and identifies a subgroup of patients with improved recurrence-free survival [7] and may also identify an histological subtype of EOC [8]. Finally, we have recently published data, demonstrating a growth regulatory role for SOX11 in *in-vitro* models of MCL [18] and EOC [8], which was further confirmed using xenotransplants in mice [19]. Together these data suggest a key growth regulatory role of SOX11 in these neoplasms and emphasize the need for functional antibodies for experimental use in various technologies, including imaging and flow cytometry applications.

Polyclonal antibodies targeting SOX11 have been widely used in immunohistochemistry on paraffin sections (IHC-P) [2,3,5,7,8,16]. However, batch to batch variations of commercially available reagents have prevented routine clinical use where standardized protocols are a prerequisite. Furthermore, there is increasing desire for expanded use of flow cytometry in immunoprofiling of tumors. This technology has already been used for several decades to confirm B cell clonality and characterize the phenotype of cellular constituents in aspirates and sections of lymphoproliferative disorders [20]. Although rarely used as the sole tool for diagnosis, studies have shown that flow cytometry is both a sensitive and specific method to identify, and in many cases, classify B cell lymphomas [21]. In contrast to IHC, flow cytometry enables quantitative measurements at the single cell level and provides the ability to analyze and quantify the co-expression of proteins in defined subpopulations, in a manner superior to immunohistochemistry [22]. However, reagents targeting SOX11 in flow cytometry have until now been lacking.

In this study, we demonstrate that the monoclonal SOX11-C1 antibody enables improved use of SOX11 as a diagnostic antigen in MCL. This antibody provides high sensitivity and improved specificity in IHC, which has enabled reclassification of previously positive HCL cases. Furthermore, the SOX11-C1 antibody allowed detection of SOX11 by flow cytometry and immunofluorescence microscopy. To assess the potential to identify rare MCL cells in blood through flow cytometry analysis, dilution experiments were performed, which permitted detection of <1% MCL cells, using SOX11 as the single discriminate antigen. In summary, the novel SOX11-C1 antibody fulfills a clinical need for increased specificity in IHC-P and extends its diagnostic utility to flow cytometry analysis.

## Results

### Generation and characterization of monoclonal SOX11 antibodies

Mouse monoclonal antibodies raised against a C-terminal part of SOX11 were generated using hybridoma technology [23]. SOX11 reactivity and lack of reactivity to the purification tag (hexahistidyl albumin binding tag, His-ABP) was assessed in ELISA and 19 clones were isolated and subcloned. Antibody isotyping revealed a high percentage of clones encoding the IgM heavy chain (68%) but clones encoding IgG could also be identified (26% IgG1 and 5% IgG2a). Concentration of selected antibodies (n = 5, all IgG1) were assessed and ranged between 1.6 and 4.6mg/ml.

**Figure 1:**
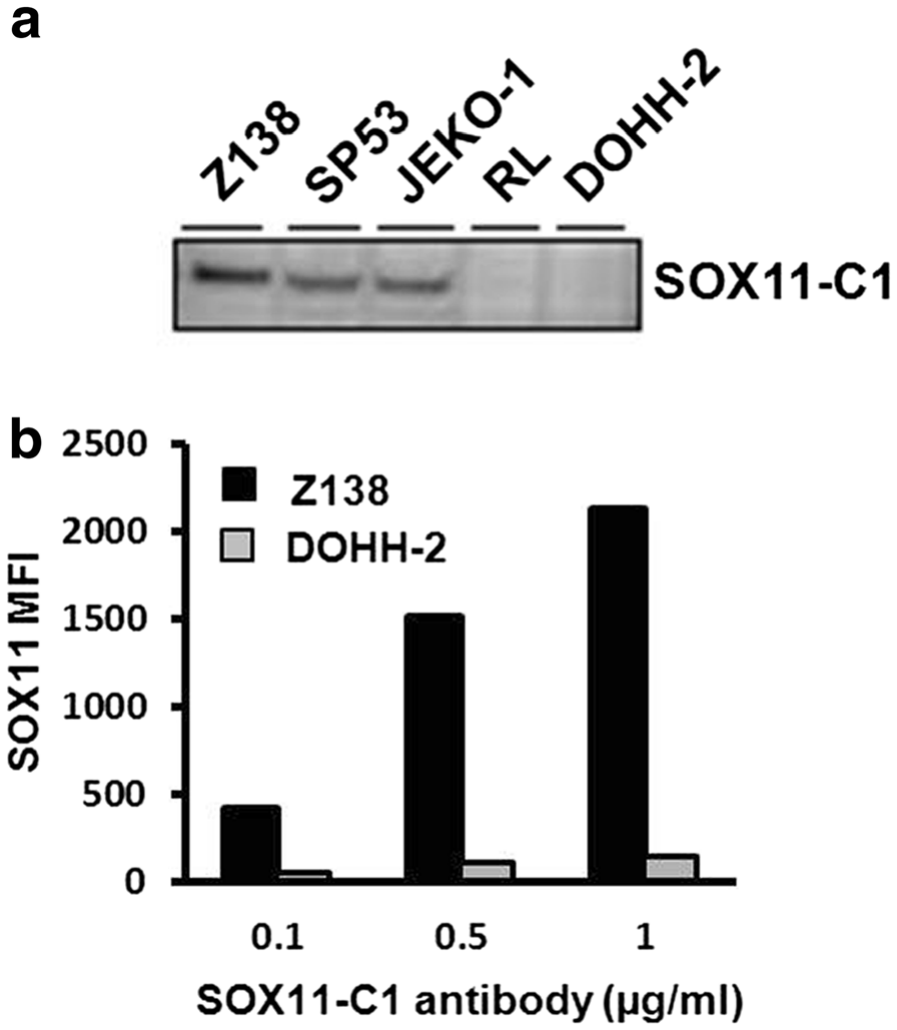
**Western blot and flow cytometry based evaluation of SOX11-C1 antibody.** (**a**) WB analysis revealed a single band at predicted size in SOX11 positive compared to negative cell lines. (**b**) Flow cytometry analysis using SOX11–C1 monoclonal antibody showed specific staining of SOX11 positive compared to negative cells lines over a range of concentrations.

The SOX11-C1 antibody was selected for further analysis based on superior performance in multiple applications, including identification of SOX11 protein at predicted size in Western blot (WB) analysis (Figure 1a) and separation of SOX11-positive (Z138) compared to SOX11-negative (DOHH-2) cell lines over a range of concentrations (Figure 1b). A competitive ELISA was used to estimate binding affinity for SOX11-C1 to approximately 0.6·10^9^ M^-1^. Sequencing of SOX11-C1 antibody revealed the use of IGKV3-2*01 F, IGKJ1*01 F, IGHV1-9*01 F, IGHD1-1*02 F and IGHJ4*01 F immunoglobulin genes according to IMGT analysis ( http://www.imgt.org).

### Immunohistochemistry-based validation of SOX11-C1

**Figure 2:**
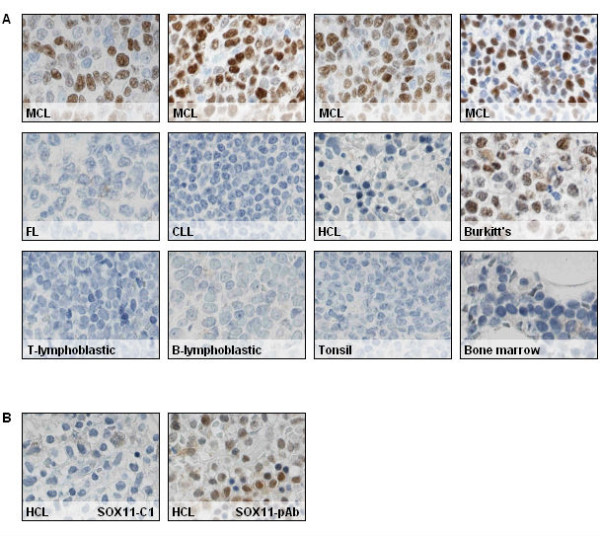
**IHC analysis of SOX11. (a)** IHC analysis using SOX11-C1 antibody showed bright nuclear staining in 100% of MCL (TMA) cases. Most other tissues including, B and T lymphoblastic lymphomas, FL, CLL, HCL, tonsil sections and non-malignant bone marrow show no evidence of nuclear SOX11 staining, except Burkitt’s lymphoma where two of four cases showed moderate nuclear staining. Representative stainings of MCL and control tissues are shown. (**b**) SOX11-C1 antibody enabled reclassification of previously positive HCL cases that lack SOX11 mRNA expression. Representative staining of SOX11 polyclonal antibody and SOX11 C1 antibody is shown.

The SOX11-C1 antibody was evaluated for routine diagnostic use in IHC-P. Identification of SOX11 protein was analyzed in a range of paraffin-embedded tissues including MCL, FL, CLL and tonsil, assembled into a TMA as previously described [1]. B and T lymphoblastic lymphomas, HCL, Burkitt’s lymphoma and non-malignant bone marrow were evaluated using whole tissue sections. Patient characteristics are summarized in Table 1. Primary MCLs showed nuclear staining (Figure 2) in all fifteen cases included in the TMA, which indicates improved [1,16] or similar [6] sensitivity compared to previous studies. The majority of cases showed strong immunostaining of the nucleus while four cases were moderately stained. A single case of a previously negative MCL was assessed, using a whole tissue section, and was confirmed to be negative. The origin of this case was confirmed using both immunophenotyping and multiplex PCR demonstrating a clonal IgH-rearrangement and a 11;14 translocation. Thus, although 100% of the cases included in the TMA tested were positive, it is evident that rare cases of SOX11 negative MCLs occur. None of the other evaluated malignant tissues, including fourteen cases of CLL, seven cases of HCL, four cases of T- and two cases of B lymphoblastic lymphoma showed nuclear SOX11 staining and in most cases also lacked background cytoplasmic staining. Additionally, non-malignant bone marrow (n = 5) and tonsil sections (n = 13) showed no evidence of SOX11-specific nuclear staining and the general background was low, in concordance with previous data [1]. Consistent with previous findings [3,16], two of four cases of Burkitt’s lymphoma showed moderate nuclear staining of SOX11. Representative SOX11 stainings of MCLs, Burkitt’s lymphoma and control tissues are shown in Figure 2. Of note, previous reports where a polyclonal antibody was used have suggested that a fraction of HCL cases are SOX11 positive [5,16]. However, using the novel SOX11-C1 antibody, none of the tested HCL cases (0/7) show nuclear staining, which is in concordance with previous mRNA data [1]. Reclassification of a single HCL case using SOX11-C1 antibody is shown in Figure 2b.

**Table 1:** Patient characteristics and SOX11 staining

**Method**	**Entity**	**N (M/F)**	**Age range (mean)**	**SOX11-C1 staining (P/T)**^**1**^
IHC-P, TMA^2^	MCL	15 (12/3)	44-82 (68)	15/15, (15/16)^5^
IHC-P, TMA	FL	15 (7/8)	49-92 (66)	0/15
IHC-P, TMA	CLL	14 (10/4)	41-82 (70)	0/14
IHC-P, WTS^3^	HCL	7 (7/0)	37-64 (51)	0/7
IHC-P, WTS	BL	4 (2/2)	3-12 (8)	2/4
IHC-P, WTS	T-lymphoblastic	4 (3/1)	13-70 (53)	0/4
IHC-P, WTS	B-lymphoblastic	2 (0/2)	14-69 (42)	0/2
IHC-P, TMA	Tonsil	13 (5/8)	5-48 (24)	0/13
IHC-P, WTS	Bone marrow	5 (3/2)	30-74 (49)	0/5
Flow cytometry	MCL	5^4^ (2/3)	50-80 (69)	5/5
Flow cytometry	FL	2 (0/2)	70-76	0/2
Flow cytometry	PBMC	n.a	n.a	0/2
Flow cytometry	Tonsil	n.a	<7	0/2

^1^Number of positive (P) cases/Total number (T) of tested cases in IHC or flow cytometry.

^2^Tissue micro array (TMA).

^3^Whole tissue section (WTS).

^4^ Includes two cases of peripheral blood.

^5^A single previously negative case was selected, tested (WTS) and confirmed to be negative in IHC.

### Flow cytometry based validation of SOX11-C1antibody

**Figure 3:**
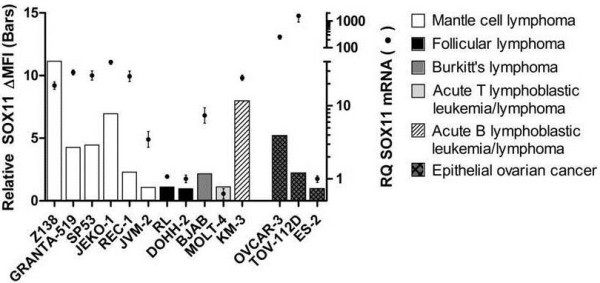
**Flow cytometry-based assessment of SOX11 status in cell lines.** Assessment of SOX11 status using flow cytometry analysis (bars) of B cell lymphoma and EOC cell lines reveal specific staining in cell lines positive for SOX11, including Z138, GRANTA 519, SP53, JEKO-1, REC-1, BJAB, KM3, OVCAR-3 and TOV-112D as assessed by RT-PCR analysis (diamonds). For each cell line, SOX11 ΔMFI was calculated as the difference between the mean fluorescence intensity (MFI) from SOX11-C1 antibody and the MFI from the control. (MFI_SOX11_ – MFI_control_). The relative SOX11 expression was calculated by scaling all data to the cell line with the lowest ΔMFI (DOHH-2).

To evaluate the performance of the SOX11-C1 antibody in flow cytometry, eleven lymphoma and three EOC-derived cell lines were used. All cell lines were evaluated for SOX11 expression (Figure 3 and Table 2) and SOX11 status (positive/negative) was defined, as previously described [18]. SOX11 positive cell lines showed strong (Z138, GRANTA-519, SP53, JEKO-1 (all MCL) and KM3 (Acute B lymphoblastic leukemia)), or moderate to weak staining (REC-1 (MCL) and BJAB (Burkitt’s lymphoma)) by SOX11-C1 antibody (Figure 3). However, SOX11 negative cell lines as JVM2 (MCL), MOLT-4 (Acute T-lymphoblastic leukemia), RL and DOHH-2 (both FL) generated only background signals, below the defined cut-off, in accordance with mRNA expression data (Figure 3 and Table 1). Fewer EOC cell lines (n = 3) were available for analysis but showed equally clear results. OVCAR-3 and TOV-112D were strongly stained compared to ES-2 in accordance with mRNA expression (Figure 3 and Table 2) and previous protein data [8]. Overall, flow cytometry analysis of SOX11 using the SOX11-C1 antibody correlated well with SOX11 mRNA levels and previously published WB data [18].

**Table 2:** Cell line characteristics and SOX11 status

**Cell line**	**Disease**	**SOX11 status**^**1**^
GRANTA-519	Mantle cell lymphoma	P
JEKO-1	Mantle cell lymphoma	P
JVM-2	Mantle cell lymphoma	N
REC-1	Mantle cell lymphoma	P
SP53	Mantle cell lymphoma	P
Z138	Mantle cell lymphoma	P
DOHH-2	Follicular lymphoma	N
RL	Follicular lymphoma	N
BJAB	Burkitt’s lymphoma	P
KM3 MOLT-4	Acute B-lymphoblastic leukemia/lymphoma Acute T-lymphoblastic leukemia/lymphoma	PN
ES-2	Differentiated ovarian clear cell carcinoma	N
OVCAR-3	Progressive adenocarcinoma	P
TOV-112D	Primary malignant adenocarcinoma	P

^1^Determined by qPCR (Positive (P) and Negative (N)) - see Methods for description (data not shown).

### Immunohistochemistry and flow cytometry-based analysis of SOX11 in tumor xenografts

**Figure 4:**
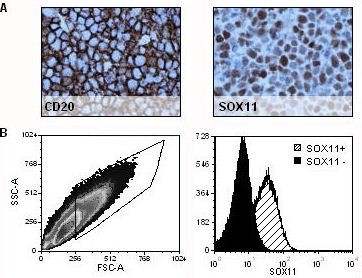
**Assessment of NSG mouse derived tumors.** Tumors established in NSG mice after intravenous injection of Z138 MCL cells were analyzed for specific SOX11 expression. (**A**) IHC-P staining showed a human B cell phenotype (CD20) and confirmed nuclear staining of SOX11. (**B**) Flow cytometry analysis separated SOX11 positive and negative cells, derived from two separate tumors established in NSG through injection of knocked (SOX11^-^) and unknocked (SOX11^+^) Z138 cells.

The potential of the SOX11-C1 antibody to identify human tumor cells in xenografts established in mice was evaluated. Z138 B cell lymphoma cells were intravenously injected in NSG mice, as previously described [19]. Single cell suspensions and tissue sections of paraffin-embedded tumors were analysed using SOX11-C1 antibody. IHC-P staining confirmed specific detection of SOX11 in CD20-expressing human tumor cells (Figure 4A) on a murine background. However, as expected, tissues with a high content of murine immunoglobulins showed extensive cross-reactivity with the secondary reagent used (data not shown). In flow cytometry, SOX11-C1 antibody specifically stained SOX11-positive cells compared to control cells (SOX11-knocked) after gating on human HLA-DR positive cells (Figure 4B).

### Immunofluorescence microscopy-based analysis of SOX11

**Figure 5:**
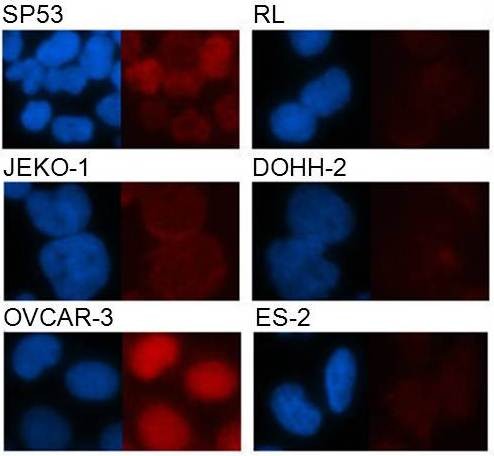
**Immunofluorescence analysis of SOX11.** Immunofluorescence microscopy analysis of SOX11 positive (SP53, JEKO-1, OVCAR-2) and negative (RL, DOHH2 and ES-2) cell lines confirm the specificity of the SOX11-C1 reagent also in imaging applications. Representative images of bright, weak and negative staining are shown. Imaging analysis reveals for the first time a nuclear localization of SOX11 also in *in vitro* cultures.

To further assess the applicability of the SOX11-C1 antibody and to determine the localization of SOX11 *in vitro*, fluorescence microscopy was used. Four B cell lymphoma cell lines expressing varying amounts of SOX11 were used and included Z138, SP53, GRANTA-519 and JEKO-1. As expected, all four SOX11 positive MCL cell lines showed nuclear staining of SOX11. Z138 and SP53 (Figure 5) showed bright nuclear staining while GRANTA-519 and JEKO-1 (Figure 5), in contrast to their flow cytometry data, were more weakly stained. None of the negative cell lines used, including RL and DOHH-2, showed nuclear staining of SOX11. In addition, specific detection of SOX11 was confirmed in OVCAR-3 (Figure 5) and TOV-112D compared to ES-2 (Figure 5). In agreement with flow cytometry data, OVCAR-3 showed the brightest staining among the EOC cell lines. The potential to visualize SOX11 in microscopy enables future experimental studies of SOX11 and co-interacting transcription factors and confirm for the first time a nuclear localization of SOX11 also *in vitro*, indicating a functional use of this transcription factor in expressing cell lines.

### Flow cytometry analysis of SOX11 in primary lymphocytes and malignant cells

**Figure 6:**
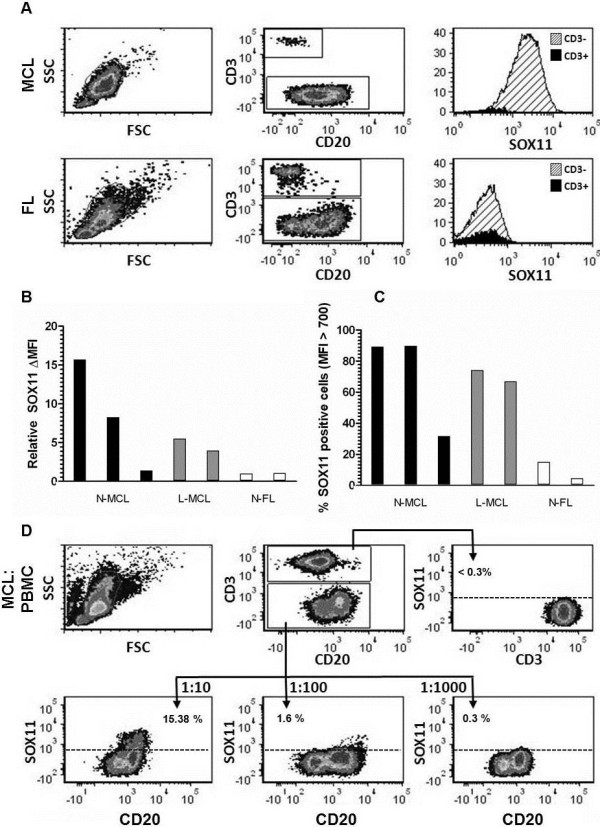
**Flow cytometry-based assessment of the detection sensitivity of SOX11 in primary MCL cells.** Flow cytometry-mediated analysis of SOX11 (**a**) showed that MCL cells were specifically stained compared to infiltrating T cells (MCL_CD3+_) and SOX11-negative malignant cells (FL_CD3-_). The specificity of the staining was assessed by comparing (**b**) the relative Δ MFI and (**c**) the fraction of SOX11 positive cells for each patient sample. Both nodal and leukemic cases of MCL were separated from FL using the SOX11 staining. (**d**) The sensitivity of the SOX11 staining was estimated by diluting (1:1, 1:10. 1:100 and 1:1000) L-MCL cells in PBMC.

The potential of the SOX11-C1 antibody to separate primary samples of MCL from negative non-expressing lymphomas and non-malignant cells, using flow cytometry was assessed. MCL cells derived from either nodal (n = 3) (N-MCL) or leukemic (n = 2) compartments were compared to nodal FL (N-FL) (n = 2) and non-malignant tissues, including PBMC and tonsil. Patient characteristics are summarized in Table 1. SOX11 intensities were defined for both malignant (CD3^-^) and non-malignant cells (CD3^+^) (Figure 6a). MCL tumor cells showed increased expression of SOX11 compared to both other lymphomas (FL_CD3-_) and non-malignant infiltrating cells (MCL_CD3+)._ This was manifested either as increased ΔMFI (Figure 6b) or fraction of SOX11 positive cells (Figure 6c). A cut-off value for SOX11 positivity was defined as SOX11_CD3-_ MFI > 700. All non-malignant tissues tested, including PBMC (Figure 6d) and tonsil (data not shown) were negative in agreement with previous data [18].

Furthermore, to assess the sensitivity of SOX11 as a discriminant antigen in flow cytometry-based assays, leukemic MCL samples were mixed with normal PBMC (ratio MCL:PBMC; 1:0; 1:1, 1:10, 1:100, 1:000 and 0:1) and analyzed (Figure 6d). Analysis of non-MCL populations showed that the SOX11-C1 antibody stained ≤0.3% of the control cells. The percentage of positive cells decreased from 65% (pure MCL) down to 48% (1:1), 15.4% (1:10), 1.6% (1:100) and 0.3% (1:1000). The data is representative of two independent MCL donors. Using SOX11 as a single discriminate antigen, malignant cells were readily detected above background down to a dilution of 1:100, i.e. a population of cells containing 1.6% MCL cells. Of note, identification of tumor cells in flow cytometry normally requires the use of multiple markers and advanced gating strategies, emphasizes the strength of SOX11 as a discriminate antigen.

## Discussion

SOX11 has recently been shown to have important diagnostic and prognostic value in MCL, EOC and glioma [1-5,7,8,24]. As the expression of SOX11 is restricted to immature neurons in the healthy adult [10], specific detection of SOX11 positive malignant cells is feasible using IHC-P in most tissues. However, previous reports, where a polyclonal antibody was used, show cytoplasmic and nuclear staining of SOX11 in FL [1,3,5,16] and HCL [5,16], respectively, despite lack of SOX11 transcription [1].

Considerable efforts are needed to standardize antibodies for clinical use in IHC-P. Reproducible detection of a specific antigen is dependent on multiple parameters, including type and concentration of fixative, incubation time, antigen retrieval, antibody concentration and detection system [25-27]. This is exemplified by the limited availability of antibodies targeting SOX11 in IHC-P; only two out of more than thirty commercially available antibodies targeting SOX11 can be successfully used in IHC-P [1,2,5,7,8,16,28]. Furthermore, batch to batch variation of polyclonal antibodies is a concern, and has limited the routine clinical use of SOX11.

To overcome these limitations, we developed a monoclonal antibody raised against a C-terminal peptide of SOX11, p-SOX11^c-term^, which has no homology to the closely related protein SOX4 [29]. This feature minimizes the possibility of cross-reactivity to SOX4 in B and T lymphocytes, which would compromise analysis of SOX11 in complex tissues. Monoclonal antibodies, such as the SOX11-C1 antibody, are consequently of great importance since they offer single epitope specificity and no batch to batch variation. Additionally, a hybridoma clone in murine or hamster cell lines enables large-scale production at low cost, as recently reviewed by Yamada [30].

We show, using the monoclonal SOX11-C1 antibody, that 100% of the MCL cases (TMA) tested presented nuclear staining of SOX11, suggesting a disease-defining property. This is in accordance with recent reports, in which SOX11 also was shown to identify rare cases of cyclin D1-negative MCLs with either typical [3] or blastoid [6] morphology. However, the rare occurrence of SOX11 negative cases, was confirmed in a single MCL case, defined by 11;14 translocation and immunophenotyping. Future investigations using large, well-defined patient series will provide reliable statistics on the infrequent occurrence of SOX11-negative MCLs. Of interest, several cases of bone marrow biopsies of HCL with previously weak nuclear staining [16] where reclassified as negative, in accordance with their lack of mRNA. Thus, this novel monoclonal SOX11-specific antibody shows high sensitivity and improved specificity in IHC-P, emphasizing its value in diagnosis and follow-up. However, future studies are needed to determine the frequency of SOX11 positive cases among primary Burkitt’s and T- and B lymphoblastic lymphomas.

As previously discussed, flow cytometry has emerged as an important clinical tool in the diagnosis and follow-up of patients after treatment. Also for intracellular antigens such as cyclin D1, successful detection of tumor cells has been shown in >90% of cases with blood involvement [31]. However, to enable assessment of cyclin D1 overexpression in patient samples, highly standardized procedures are required [32]. To determine whether the SOX11-C1 antibody allow intracellular detection of SOX11 in flow cytometry, we performed a proof-of-principle assay and analyzed well-defined lymphoma and EOC cell lines. In agreement with mRNA expression and previous WB data [18], SOX11 positive cell lines where distinguished from negative ones in all cases, which implies high specificity and sensitivity of flow cytometry analysis of SOX11. Of note, SOX11 was detected in KM3, an acute B lymphoblastic leukemia cell line, and support previous findings of SOX11 positive B lymphoblastic lymphomas using IHC-P [16]. Also in a more complex setting, using a single cell suspension derived from a tumor established by intravenous injection of Z138 MCL cells into NSG mice, SOX11-C1 was able to distinguish SOX11-positive from negative samples.

To evaluate the potential clinical use of flow cytometry in patients with disseminated SOX11-positive disease, dilution experiments where performed. We demonstrate that malignant cells, comprising as little as 1% of the total lymphocyte population are detectable, using SOX11 as the sole discriminant antigen. Future attempts to replicate these findings in patients with various degree of disseminated disease and in combination with immunophenotyping would be of great interest. In combination, the simplicity of the flow cytometry protocol and the disease-defining property of the SOX11 antigen suggest its future use in both routine diagnostics and for detection of circulating tumor cells during patient follow-up. For potential assessment of minimal residual disease, careful comparison with IGH-PCR needs to be performed as, flow cytometry has shown to be inferior to IGH-PCR at follow-up after treatment [33]. This difference in sensitivity may be partly related to the clearance of B cells upon rituximab (anti-CD20) treatment, thereby increasing the relative sensitivity of IGH-PCR based detection.

## Conclusions

There is an unmet clinical and experimental need for specific and sensitive analysis of SOX11 in relation to SOX11-positive tumors, including MCL and EOC. The novel SOX11 monoclonal antibody, SOX11-C1, offers improved functionality, such as (i) immunofluorescence-based investigations (ii) improved specificity in IHC-P, enabling more sensitive and specific diagnosis and (iii) flow cytometry-based detection, which is potentially feasible in aspirates from MCL and for detection of circulating tumor cells.

## Methods

### Generation of monoclonal antibodies targeting SOX11

A C-terminal peptide of SOX11 (Table 3), referred to as p-SOX11^c-term^ and previously defined in the Human Protein Resource project [16,34], was used as immunogen. C57BL/6 mice were immunized and hybridomas were produced by fusion of splenocytes and mouse myeloma SP2/0 cells, using standard procedures [23]. Heavy-chain immunoglobulin use, antibody production and SOX11 reactivity was assessed in 2800 individual clones, using ELISA. Antibodies were purified on protein A columns from culture supernatants. Concentrations were determined at OD_280_ and purity was assessed on SDS-PAGE.

**Table 3:** SOX11-specific peptide used for immunization

**Antigen**	**Alias**	**Sequence**^**1**^
SOX11	p-SOX11^c-term^	EDDDDDDDDDELQLQIKQEPDEEDEEPPHQQLLQPPGQQPSQLLRRYNVAKVPASPTLSSSAESPEGASLYDEVRAGATSGAGGGSRLYYSFKNITKQHPPPLAQPALSPASSRSVSTSSS

^1^Sequence was originally derived from the HPR project as previously described [35].

### Subcultivation of cell lines and assessment of SOX11 mRNA expression

A number of lymphoma/leukemia and EOC cell lines, including Z138, GRANTA-519, JEKO-1, SP53, REC-1, JVM-2, RL, DOHH-2, BJAB, KM3, MOLT-4, ES-2, OVCAR-3, and TOV-112D, were purchased from ATCC or DSMZ (or when not available, received as kind donations, see acknowledgement). Lymphoid cell lines were cultured in RPMI-1640 (Thermo Scientific HyClone, Logan, UT, USA) supplemented with 10% fetal bovine serum (Invitrogen, San Diego, CA, USA) and 2 nM L-glutamine (Invitrogen). EOC cell lines where cultured in either McCoy’s (Thermo Scientific HyClone) + 10% FBS and 2 mM L-glutamine (ES-2), DMEM high glucose (Thermo Scientific HyClone) + 10% FBS and 2 mM L-glutamine (TOV-112D) or RPMI-1640 + 20% FBS + 2 mM L-glutamine + 0.01 mg/ml insulin (Sigma-Aldrich) (OVCAR-3). All cell lines were kept under standard conditions (5% CO_2_, 37°C). The relative quantity (RQ) of SOX11 mRNA in these cell lines was measured with real time-quantitative PCR (RT-PCR), as previously described [18]. The RQ is calculated as 2^-(ΔΔCT(SOX11-GAPDH))^ and cell lines with a ΔC_T (SOX11+RT, SOX11-RT)_ > |2| were defined as SOX11 positive. Error bars have been calculated using the standard error value with 95% confidence interval.

### Evaluation of the SOX11-C1 antibody in Western blot using B cell lymphoma cell lines

Protein lysate (30 μg) obtained from B cell lymphoma cell lines were run on a NuPAGE 10% Bis-Tris gel (Invitrogen). Proteins were blotted onto a PVDF membrane with the iBlot® Dry Blotting System (Invitrogen) and the membrane was blocked in 5% milk/PBS. SOX11 expression was assessed using SOX11-C1 at a final concentration of 1 μg/ml in 5% milk/PBS. A primary antibody targeting GAPDH was used as loading control and a HRP labeled rabbit anti-mouse antibody (Dako, Glostrup, Denmark) was used for detection. Labeled proteins were visualized using SuperSignal West Femto Max Sensitivity Substrate (Pierce Biotechnology, Rockford, IL, USA), and images retrieved using a CCD camera (FluorSMax, Biorad). Proteins were quantified, using the Volume Rect Tool (Quantity One, Biorad), and the relative volume of the background was set to 1.

### Flow cytometry analysis of SOX11 in B cell lymphoma and EOC cell lines

Lymphoma/leukemia and EOC cell lines were fixed in 4% paraformaldehyde (PFA) (Thermo Scientific) for 10 minutes. The cells were subsequently permeabilized in PBS supplemented with 5% FCS and 0.5% Triton X-100 (Sigma Chemical Co., St Louis, USA). SOX11 was stained using SOX11-C1 antibody at 1 μg/ml for 16 h. Primary antibody and unstained control cells were detected using goat anti-mouse-APC (BD Bioscience, San Jose, CA, USA). All samples were analyzed on FACS Canto II (BD Bioscience) and FCS Express 4 Flow Research Edition (De Novo Software, Los Angeles, CA USA) was used for data analysis.

### Sequencing of immunoglobulin genes coding for the SOX11-C1 antibody

The immunoglobulin genes coding for SOX11-C1 were sequenced in both heavy and light chain variable regions (VH, VL) according to standard procedures (Absea Biotechnology Ltd, Beijing, China).

### Immunohistochemistry analysis using SOX11-C1 antibody

SOX11-C1 monoclonal antibody was tested on a tissue microarray (TMA) consisting of fifteen MCL, fourteen CLL, fifteen follicular lymphomas (FL), two Burkitt’s lymphomas and thirteen tonsil sections. Additionally, whole sections of one MCL, two Burkitt’s lymphomas, seven HCL, two B-lymphoblastic lymphomas, four T-lymphoblastic lymphomas and five normal bone marrows were tested. Tumors developing after intravenous injection of Z138 in NOD SCID gamma (NSG) mice were prepared, as previously described [19]. Bone marrow biopsies from HCL were fixed in 10% formalin and decalcified until softened in a solution of ethanol, nitric acid and chromium trioxide at room temperature, while bone marrows obtained from healthy donors or B-lymphoblastic lymphomas were decalcified in a solution of EDTA at 37°C. All samples were routinely processed and embedded in paraffin for tissue conservation. Antigen retrieval was performed using the PT-LINK system (Dako) at pH 9. Staining was performed using SOX11-C1 (1-2ug/ml). For detection, Envision + System HRP (Dako) was used on a DAKO autostainer Plus (Dako). Staining of a single HCL case using a polyclonal reagents targeting SOX11, was performed as previously described [3,16].

### Affinity estimations using competitive ELISA

The affinity of the SOX11-C1 antibody to its antigen was estimated, using a competitive setup in ELISA. Briefly, a high binding microtiter plate was coated with p-SOX11^c-term^ (10 ng/well). After blocking, a fixed concentration of SOX11-C1 (0.1 μg/ml) was pre-incubated with the antigen, p-SOX11^c-term^, at an optimized concentration range (0–100 nM) for 1 hour. The amount of unbound antibodies in solution was measured with ELISA. The dissociation constant (K_D_) was estimated to equal the antigen concentration at 50% signal inhibition.

### Image analysis of SOX11 using fluorescence microscopy

Slides for image analysis were prepared by (i) immobilization of lymphoma cells (40,000 cells/slide) onto a glass slide, using a cytospin (Shandon, Pittsburgh, USA) or (ii) cultivation of EOC cells in eight-well chamber slides (Nalge Nunc International, Rochester, NY, USA). Cells were fixed in 4% PFA for 10 min followed by permeabilization (PBS supplemented with 5% FCS and 0.5% Triton X-100, (Sigma Chemical Co)). Cells were stained with SOX11-C1 antibody (1 μg/ml, overnight) followed by a goat anti-mouse IgG-ALEXA 568 reagent (Invitrogen). The nuclei were counterstained with DAPI (Invitrogen/Dynal) and mounted with Pro Long Gold anti-fade medium (Invitrogen). Images were captured (exposure time; DAPI 40 ms, TX RED 2 s) using a fluorescence microscope (Nikon Eclipse 80i, Nikon Instrument Inc. NY, USA) equipped with Nis Element BR (Nikon).

### Flow cytometry analysis of SOX11 in NSG mice-derived tumors

Tumors were established in female NSG mice (The Jackson Laboratory, Sacramento, California, USA, (bred at Lund University for multiple generations)) by intravenous injection of Z138 cells (0.5 × 10^6^ cells) carrying SOX11-specific (SOX11-knocked) or scrambled (control) shRNA sequences. Animals were sacrificed when presenting symptoms of disease, as previously described [18]. All animal experimentation was strictly performed in accordance with the regulations of the Lund University Ethics Committee (Dnr M229-09).

Single cell suspensions from tumors developing in the ovaries were prepared. Cells were stained in two separate tubes, i.e., (i) extracellular with HLA-DR-APC (BD Biosciences) or (ii) stained with SOX11-C1 after fixation and permeabilization, as described above. FSC and SSC gating strategy was based on HLA-DR positive cells as a marker for human B cells. The same gating strategy was used to assess SOX11 expression in the complex mixture of cells.

### Flow cytometry analysis of SOX11 in primary B cell lymphomas

Peripheral blood mononuclear cells (PBMC) from two leukemic MCL (L-MCL) patients and two non-malignant blood donors were isolated by Ficoll (VWR) gradient centrifugation, according to standard procedures. Furthermore, single-cell suspensions from three MCL, two FL, and two non-malignant tonsil biopsies were prepared by dissection of tissue, with resultant diffusion of malignant cells into the media (RPMI-1640). Malignant cells (L-MCL) were mixed with PBMC at the following ratios; 1:0 1:1, 1:10, 1:100, 1:1000 and 0:1. For each sample, 500,000 cells were stained with SOX11-C1 antibody as described above but with the following modifications: after addition of goat anti-mouse-APC the samples were blocked with mouse IgG (Jackson ImmunoResearch Laboratories Inc., West Grove, PA, USA) for 30 minutes followed by staining with CD3-PE (Invitrogen) and CD20-FITC (Dako).

## Abbreviations

BL: Burkitt’s lymphoma; CCND1: Cyclin D1; CLL: Chronic lymphocytic lymphoma/leukemia; ELISA: Enzyme linked immunosorbent assay; FL: Follicular lymphoma; HCL: Hairy cell leukemia; IHC-P: Immunohistorychemistry using paraffin embedded tissue; LG: Lymph gland; MCL: Mantle cell lymphoma; IGH: Immunoglobulin heavy chain; N: Negative; NSG: NOD SCID gamma; PB: Peripheral blood; PBMC: Peripheral blood mononuclear cells; P: Positive; VH: Variable heavy chain; TMA: Tissue micro array; VL: Variable light chain; WB: Western blot; WTS: Whole tissue section.

## Competing interests

A patent has been filed on the diagnostic, prognostic and therapeutic use of SOX11 in B cell lymphomas and EOC. The authors declare that they have no competing interests.

## Authors' contributions

LN performed the experimental work and wrote parts of the manuscript. UA generated murine tissues for SOX11 assessment as well as analysed the data and helped with the completion of the manuscript. MD and MJ provided IHC and primary tumor material and helped with the completion of the manuscript. CB was involved in the design of the study and the completion of the manuscript. SE was responsible for the design of the study, interpretation of data and writing of the manuscript. All authors read and approved the final manuscript.

## Pre-publication history

The pre-publication history for this paper can be accessed here:

http://www.biomedcentral.com/1471-2407/12/269/prepub
